# How to Choose the Superconducting Material Law for the Modelling of 2G-HTS Coils

**DOI:** 10.3390/ma12172679

**Published:** 2019-08-22

**Authors:** Bright Chimezie Robert, Muhammad Umar Fareed, Harold Steven Ruiz

**Affiliations:** Department of Engineering and Leicester, Institute for Space & Earth Observation Science, University of Leicester, Leicester LE17RH, UK

**Keywords:** superconducting coil, alternating current (AC) losses, superconducting material law

## Abstract

In an attempt to unveil the impact of the material law selection on the numerical modelling and analysis of the electromagnetic properties of superconducting coils, in this paper we compare the four most common approaches to the E-J power laws that serve as a modelling tool for the conductivity properties of the second generation of high-temperature superconducting (2G-HTS) tapes. The material laws considered are: (i) the celebrated E-J critical-state like-model, with constant critical current density and no dependence with the magnetic field; (ii) the classical Kim’s model which introduces an isotropic dependence with the environment magnetic field; (iii) a semi-empirical Kim-like model with an orthonormal field dependence, $J_{c}\left(B\right)$, widely used for the modelling of HTS thin films; and (iv) the experimentally measured E–J material law for SuperPower Inc. 2G-HTS tapes, which account for the magneto-angular anisotropy of the in-field critical current density $J_{c}\left(B;\theta \right)$, with a derived function similar to Kim’s model but taking into account some microstructural parameters, such as the electron mass anisotropy ratio (γ) of the superconducting layer. Particular attention has been given to those physical quantities which within a macroscopic approach can be measured by well-established experimental setups, such as the measurement of the critical current density for each of the turns of the superconducting coil, the resulting distribution of magnetic field, and the curve of hysteretic losses for different amplitudes of an applied alternating transport current at self-field conditions. We demonstrate that although all these superconducting material laws are equally valid from a purely qualitative perspective, the critical state-like model is incapable of predicting the local variation of the critical current density across each of the turns of the superconducting coil, or its non-homogeneous distribution along the width of the superconducting tape. However, depending on the physical quantity of interest and the error tolerance allowed between the numerical predictions and the experimental measurements, in this paper decision criteria are established for different regimes of the applied current, where the suitability of one or another model could be ensured, regardless of whether the actual magneto angular anisotropy properties of the superconducting tape are known.

## 1. Introduction

In recent years, advances in the development of high-temperature superconducting coils with rare-earth barium-copper oxide (REBCO)-coated conductors have drawn significant attention by the community of researchers in applied superconductivity, thanks to the vast progress in the technology of thin films that has enabled the fabrication of the second generation of high-temperature superconducting (2G-HTS) tapes in the past decade. Thence, the formulation of modelling tools for describing the electromagnetic and thermal properties of such 2G-HTS tapes and their use in high-power-density coils for applications such as superconducting fault current limiters [1,2,3], transformers [4,5], power generators [6,7,8], motors [9,10,11,12], energy storage systems [13,14,15], permanent magnets [16,17], and magnetic imaging machines [18,19] is currently a motivating force of intensive research due to the inherent complexity of the material law that governs the electrical properties of the superconducting compound, the computational challenges that are imposed by the large cross-sectional aspect ratio of the 2G-HTS tape, and ultimately, the actual size of the coils that need to be modelled before investing in usually large and customised cryogenic facilities for their experimental testing.

Moreover, multiple publications have already reported that the electrical properties of the large majority of 2G-HTS tapes can be strongly influenced by the intensity and direction of externally and self-induced applied magnetic fields [20,21,22], although no strong agreement has been reached in terms of the material law that governs their critical current density properties, as widely different material laws can render similar results depending on the physical quantity being studied [22,23,24]. In this sense, the simulation of the electromagnetic behaviour of 2G-HTS coils presents serious challenges, especially in conditions where one needs to consider the in-field dependence of the critical current, $I_{c}$, this in terms of the direction and intensity of the magnetic field per coil-turn, as pointed out in early experiments measuring the magnetic field distribution and AC loss of HTS thin films in superconducting coils [25,26]. Nonetheless, regardless of the 2G-HTS tape being used, certain consensus has been reached in terms of describing the current-voltage characteristics of all type-II superconductors as a power law, $V{\left(I/I_{c}\right)}^{n}$, with n≫1, which from the computational point of view renders the well-known form of the material law for the electric field, also called the E-J power law, $E\left(J\right)=E_{0}\cdot J/J_{c}\cdot \left(|J\right|/J_{c}{)}^{n-1}$, which in a local but macroscopical approach allows solution of the Maxwell equations inside the superconducting domains within diverse mathematical formulations, for a large range of experimental measurements [16,23,27,28,29,30,31,32,33,34,35,36,37,38]. Here, $J_{c}$ is the critical current density of the 2G-HTS tape defined within the standard electric field criterion $E_{0}$ = 1 μ V/cm, which is commonly measured under direct current–voltage measurements with I-V curves typically showing power factors n>20. However, most of the already conducted research has been focused either on the simplification of numerical models to enable faster computational algorithms for relatively large or complex geometries or on the reproduction of specific experimental evidence, such as the hysteresis losses, magnetisation curves, or the measurement of the magnetic field at specific positions that are not necessarily close to the superconducting domains, where changes in the amplitude of the critical current density can be more notorious. In fact, because of the great complexity associated with the experimental measurement of the critical current density at each turn of a superconducting coil, and given the relatively large uncertainty in the reproducibility of the experimental measurements caused by the non-homogeneity of the electric field inside the superconducting tape (both across its length and along its width), computational models are also commonly used to estimate the actual critical current density of the coils. This is done by matching the numerical results of a particular model with any specific physical quantity that can be measured by further experimental methods. However, this does not resolve the problem of the material law selection, but opens some central questions, such as: “Are computational modellers using a sound material law for the superconducting tapes?” and, “Is this material law valid for the study of any other macroscopic electromagnetic quantity?”

Thus, for this Special Issue of MDPI’s *Materials*, we present a comprehensive study of the impact of the E-J material law selection on the electromagnetic properties of superconducting racetrack coils, using the H-formulation as a benchmark [27,28] for solving the partial differential equation (PDE) system of Maxwell equations and the four most popular material law models for type-II superconductors, namely: (i) a simplified critical state (CS)-like-model [23,39]; (ii) the classical Kim’s model [40,41]; (iii) an empirical Kim-like model with orthonormal field dependence for SuperPower Inc. [42] SCS4050 2G-HTS tapes [33]; and (iv) the generalised form of the critical current density with magneto angular anisotropy measured on the SCS4050 tapes [20]. The paper is organised as follows. In Section 2, we outline the main characteristics of the material law models considered in this study and introduce the geometry and other physical conditions relevant to the solution of this problem. All the aforementioned material laws can be implemented into any finite element formulation for solving the local Maxwell equations (i.e., within the superconducting domains), which obviously will have to render to an univocal solution where the physics richness of the problem does not lie within the mathematical formulation used, but on the invoked material laws. In this sense, a detailed review on how these formulations can be numerically implemented is not within the scope of this paper; instead, all the necessary parameters and conditions that enable the reproducibility of our results are disclosed here. Then, in Section 3, we discuss the main findings from our numerical results, where similarities and differences between the diverse material laws are highlighted in terms of: (i) the analysis of the local distribution of current inside different turns of the superconducting coil; (ii) the intensity of the magnetic field near and over its innermost turn, this being the coil turn that is more prone to being affected by the self- and mutually induced currents when $J_{c}$ has a strong dependence with the magnetic field; and (iii) the curve of AC losses of the overall system when the REBCO layer of the 2G-HTS tape is modelled as a magnetically isotropic or anisotropic layer. Finally, in Section 4 we summarise the main conclusions of this study, which ultimately are aimed to help the HTS computational modellers in making sensible decisions about how to choose the superconducting material law for 2G-HTS coils, especially when not only a physical quantity needs to be quantified to match with experimental evidence, but in situations where additional consideration must be given to the computing time demanded.

## 2. Superconducting Material Law Models

In order to compare the impact of different material law models on the numerical modelling of 2G-HTS coils, below we consider a two-dimensional approach based on the celebrated H-formulation, which can be easily implemented in COMSOL Multiphysics [43]. As a benchmark case, we assumed the cross section of a 20-turn racetrack coil wound on a 5 cm midwidth former with a SuperPower Inc. (Schenectady, NY, USA) SCS4050 tape [42], with a 4 mm width and overall thickness of approximately 0.1 mm, with a self-field critical current density (i.e., the critical current density measured in the absence of external magnetic field) measured for long linear sections of $I_{c0}=114$ A, within the standard 1 μ V cm ${}^{-1}$ electric field criterion at which $E_{c}$ = 1 × 10${}^{-4}$ Vm ${}^{-1}$ [20,44]. This tape is composed by a type-II superconducting thin film made of Yttrium barium copper oxide (YBCO), with an aspect ratio of 1μ m × 4 mm, which was then split within our computations into at least 4×115 finite elements, enabling a sufficiently high resolution for the discernment of changes in the flux front profiles and the distribution of current density along and across each of the coil turns. This YBCO layer is fabricated by metal organic chemical vapour deposition (MOCVD) over a 0.2μ m buffer of heteroepitaxial layers, deposited by sputtering on a Hastelloy C-276 substrate of 50μ m, and then coated at the top and bottom by a thin Ag layer of ∼2 μ m, which provide better and more uniform electrical contact with the Cu electro-thermal stabiliser layers of 20μ m thickness each that are deposited at the top and bottom heterostructures of the 2G-HTS tape. Within the numerical scope of this paper, we neglected the thickness of the buffer layer, as all the calculations presented here are at applied currents under the critical threshold $I_{c0}$ and the materials properties are considered to be homogeneous along the entire length of the 2G-HTS tape used, and this is an ansatz that has been already demonstrated to be a reasonably valid approach for reproducing a wide range of experimental evidence [16,28]. However, given the enormous aspect ratio of the YBCO layer and the circumambient layers, including the outer domain that aims to mimic the electric free field condition for solving Faraday’s law (the latter usually called by modellers as the “air” domain), each of the Cu, Ag, and substrate layers were split into 4, 2, and 6 finite elements across their respective thickness, times 115 rectangular elements along their width, therefore matching with the mesh of the YBCO layer. In addition, we implemented a mapped triangular mesh of approximately 60,000 distributed finite elements, emulating an “air” domain of about 1200 cm ${}^{2}$ including the 0.2-mm-thick electrically insulating kapton layers between the coil turns. Then, thanks to the 2D axisymmetrical geometry of the racetrack coil, only one side of the coil needs to be modelled, for which a Dirichlet boundary condition $\left(H_{x}=0\right)$ was imposed at the centre axis of the racetrack former. Finally, for the sake of providing sufficient clarity on the physical effects derived, by the use of different material laws describing the E-J superconducting properties, only self-field conditions are considered in this study, with the racetrack coils subject to an alternating current source (AC) which obeys the function $I_{tr}=I_{a}\sin\left(\omega t\right)$.

The power law of the E-J material law in its most generic form,

(1) $$E\left(J\right)=E_{0}\cdot \;\frac{J}{J_{c}}\cdot {\left(\frac{\left|J\right|}{J_{c}}\right)}^{n-1}\;,$$

can be then synthesised within the four most common approaches for the behaviour of the critical current density, $J_{c}$, shown in Table 1, and all of them are within the isotropic hypothesis of parallelism between the vectors E and J, in principle valid for the symmetry of our problem due to the perpendicularity between the vectors *B* and *J*. Likewise, for numerical purposes, it would be possible to assume the exponent *n* to be sufficiently large to have a sharp increment in the electric field when $I>I_{c0}$, which allows neglect of the thermal agitation of vortices and other statistical effects on the conventional magneto-quasi-steady (MQS) approach [39], and therefore emulation of Bean’s classical statements [45,46] for the critical-state model [23]. However, in practical cases where finite differences in the homogeneity properties of the superconducting material need to be averaged, as it is in the case for 2G-HTS tapes, and Equation (1) can still be used as long as the parameters $I_{c0}$ and *n* are experimentally measured. For this reason, we emphasise that the 2G-HTS considered for this study corresponds to a specific product of one of the major superconducting tape manufacturers, SuperPower Inc., whose SCS4050 [42] tape is probably one of the most common tapes used by their customers, and on which these properties have been extensively measured and verified by many research groups, including the magneto-angular anisotropy characteristics of $J_{c}$ [20].

Thus, with $I_{c0}=114$ A and the exponent n=30.5, the first of the material law models to be implemented was the so-called critical-state-like model, $C_{M}$ (see Table 1), where the occurrence of current density profiles is related to a magnetic diffusion process that takes place when the local condition for the isotropic critical-state $J\leq J_{c0}\hat{E}$ is violated, where $\hat{E}$ is the unit vector that defines the direction of the electric field E, with $J=J_{c0}$ if E≠0. Note that within a purely theoretical framework, this macroscopic model can be seen as a general one, as it allows the main physics features derived from the electromagnetic properties of type-II superconductors within Bean’s approach to be captured [45,46]. However, in most practical applications of type-II superconductors including the 2G-HTS tapes, it is well known that in the manufacturing of superconducting materials, achieving widely homogeneous electrical properties is something that cannot be assured ad hoc. This is mainly due to the granular properties that these kinds of materials commonly have, and the possibility of having impurities within the structure of the superconductor (i.e., having added materials or alloys with non-superconducting properties during the fabrication of the HTS material). This situation, which is not necessarily unfavourable for the actual use of superconducting materials, is what gave rise to the flux creep concept in the early 1960s, where the occurrence and motion of local profiles of current density predicted by Bean was not only caused by the thermally activated motion of flux structures (vortices) proposed by Anderson [48], but to the local Lorentz force between collective groups of vortex lines whose intensity depends on the microstructure of the HTS material, and which can be averaged within the semi-empirical parameters $B_{0}$ and α introduced by Kim [40,41]. These parameters can be obtained from the the aforementioned I-V measurements, either under self-field conditions or externally applied magnetic fields, the first is designated to the classical version of Kim’s model, $K_{M1}$ (Table 1), and the latter to Kim-based models where better fitting functions accounting for the experimental measurements have been found. This is the case of the empirical Kim-like model $K_{M2}$ (Table 1) originally introduced by Thakur et al. [47], which has been regularly used for the modelling of 2G-HTS tapes from different manufactures, including the SCS4050 tape made by SuperPower Inc. [33], where $B_{\|}$ and $B_{⊥}$ are the local orthonormal components of the magnetic flux density, being parallel or perpendicular to the wider surface of the 2G-HTS tape. However, in collaboration with the EPEC superconductivity group at the University of Cambridge in the UK, a more general function for the in-field critical current density of 2G-HTS tapes with magneto-angular anisotropy has been recently introduced by Ruiz [20], whose model accounts not only for the flux creep parameters derived by Kim’s approach [40] (i.e., the microstructure parameters $B_{0}$ and α in Table 1), but extends this function to incorporate Blatter’s angular anisotropy factor $\epsilon_{\theta }$, which is a function of the electron mass anisotropy ratio (γ) of the REBCO layer and the angular direction θ of the local magnetic field. Remarkably, this model is capable of reproducing the full magneto-angular anisotropy properties of the critical current density, $J_{c}\left(B,\theta \right)$, for a broad set of market-available 2G-HTS tapes [20,21], currently manufactured by companies such as SuperPower Inc. [42], American Superconductor [49], Shanghai Superconductor Technology Co., Ltd. [50], and SuperOx [51]. In this sense, in this paper we called this model $R_{M}$, which completes the full set of material laws whose range of validity and scope will be critically analysed in the following section.

## 3. A Crude Analysis of the Material Law Derivatives

By assuming that the superconducting coil former lies on the zx-plane, the 2D axial symmetry commonly assumed for the modelling of long superconducting racetrack coils implies that, the components of the magnetic field inside each of the turns of the superconducting coil must lie on the “xy-plane”, $\left[H_{x},\;H_{y}\right]$, orthonormal to the direction of the flux of current density, $J_{z}$, and with the vector of electric field pointing towards the same direction, with the E-J power law in Equation (1) governing the electromagnetic properties of the REBCO layer inside the 2G-HTS tape, and all the other “normal” layers obeying Ohm’s law, $E_{z}=\rho J_{z}$. Notice that Ohm’s law is not being constrained to a threshold value of the current density, as it is the case for the E-J power laws shown in Table 1, what represents the main macroscopical difference between “normal” and “superconducting” materials from the electromagnetic point of view, where Maxwell equations are univocal. Thus, Faraday’s law, $\nabla \times E=-\partial_{t}B$, is locally solved via the defined electrical field condition for the different materials, whilst simultaneously, Ampère’s law, ∇×B=J, is globally solved by the coupling equation of the so-called H-formulation, $\nabla \times \left(\rho \nabla \times H\right)+\mu \partial_{t}H=0$. Note that in order to solve this PDE system, a couple of further ansatz for the material laws need to be taken first, which although are of common practice in applied superconductivity, are still of great importance as they complete the full definition of material laws that are invoked in the electromagnetic modelling of superconducting heterostructures such as 2G-HTS tapes. For instance, the reader might have noticed that within the common use of the H-formulation, a magnetically isotropic and homogeneous behaviour for the magnetic permeability of the different materials is being assumed (μ), such that the magnetic flux density vector B can be written in terms of the magnetic field vector H, as B=μH, where μ can approach the magnetic permeability of vacuum $\left(\mu_{0}\right)$ for all domains in the system to be modelled, as long as the different materials involved behave as non-dispersive (frequency-independent) mediums, with non-magnetic intrinsic properties in their “normal” state. This assumption can be carried out for the REBCO layer in its “superconducting” state, as long as the frequency of the external excitations (i.e., the applied transport current or any external magnetic field) is assumed as constant and it does not change the microstructural properties of the superconducting material [47]. Moreover, as in our definition of Ampère’s law, we have invoked the MQS approach [52], where the displacement current densities $\delta_{t}D$ are much smaller than J, disappearing in a first-order treatment, it is possible to assume that for uniform and slow sweep rates of the external excitations, the transient variables of electric field E and electrical resistivity ρ are both small and proportional to $\dot{B}=\partial B/\partial_{t}$, whereas $\ddot{B}$, $\dot{E}$, and $\dot{\rho }$ are negligible. Therefore, defining sufficiently small time steps in the computation of the PDE system, Ampère’s law can be rewritten as $\nabla^{2}H-\mu \sigma \partial_{t}J=0$, with approximate integrability condition ∇·J≃0, where the electrical resistivity function $\rho \left(J\right)=\sigma^{-1}$ plays the role of a nonlinear and possibly nonscalar resistivity in the case of the REBCO layer in the superconducting state, that is, $\sigma^{-1}=E_{0}/\left|J\right|\cdot {\left(\left|J\right|/J_{c}\right)}^{n}$, with the critical current density $J_{c}$ defined in Table 1, and a constant value within the isotropic version of Ohm’s law, J=σE, for all remaining “normal” materials.

**Figure 1:**
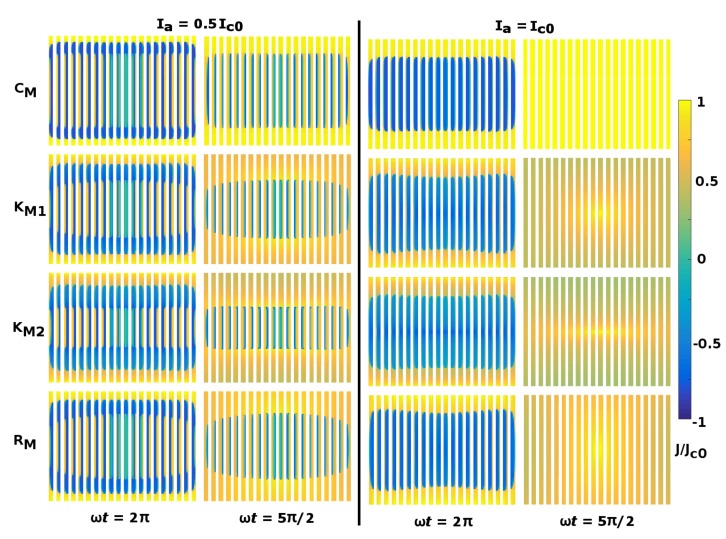
Local profiles of normalized current density $J/J_{c0}$ inside each of the superconducting layers for a 20-turn second generation of high-temperature superconducting (2G-HTS) racetrack coil (not to scale), with the innermost turn being the leftmost 2G-HTS domain (layer) shown in each of the displayed subplots, and with applied AC currents of amplitude $I_{a}=0.5\;I_{c0}$ (left pane) and $I_{a}=I_{c0}$ (right pane), conditioned to the HTS material laws introduced in Table 1. For an easy visualisation of the current-density distributions, results are shown by artificially expanding the thickness of the REBCO layer inside the 2G-HTS tape, as no electrical current flows in any of the other composite layers.

Thus, based on the aforementioned physics framework for the material laws of “superconducting” and “normal” materials, and on which the scope of this study must be understood, below we present the main observations derived from the critical analysis on the use of one or another material law, for the modelling of the superconducting properties of the REBCO layer in a 2G-HTS racetrack coil. We pay special attention to measurable (experimental) quantities such as the critical current density, the magnetic field, and the AC losses, all from the local dynamics of the flux of current density across the cross section of the superconducting coil (Figure 1). We encourage the reader to download the high-resolution figures attached to this paper, such that more enhanced visualisation of the figures can be achieved where applicable.

### 3.1. Dynamics of Flux Front Profiles: A Qualitative Approach

Firstly, we present a qualitative comparison between the four choices for the superconducting material law shown in Table 1, based on which it is possible to analyse how the distribution of current-density profiles change in time for different $J_{c}$ functions. It is worth mentioning that a full analysis of the time dynamics of flux front profiles inside the HTS domains under the $R_{M}$ model (excluding the $C_{M}$, $K_{M1}$ and $K_{M2}$ models) has already been presented elsewhere [27], where equivalent high meshing considerations were applied in order to obtain a realistic account of the local and global electromagnetic properties of racetrack coils, and where well-defined regions with clear patterns obeying the occurrence of magnetisation currents, transport currents, and flux-free cores can be envisaged for the whole hysteretic behaviour of the racetrack coil with applied transport currents $I_{tr}=I_{a}\sin\left(\omega t\right)$, of amplitudes ranging between $I_{a}=0.1I_{c0}$ and the self-field threshold value $I_{a}=I_{c0}$. Thus, as the main physical features encountered in the evolution of the flux front profiles for the different material law models have been found to be essentially the same as those reported in [27], for the sake of simplicity, here we display the profiles of current density for only moderate and high applied currents, $I_{tr}=I_{a}\sin\left(\omega t\right)$, with amplitudes $I_{a}=0.5I_{c0}$ and $I_{a}=I_{c0}$, respectively. These are shown for the two steady steps representing either the self-field condition, $I_{tr}\left(t\right)=0$, or the positive peak of current, $I_{tr}\left(t\right)=I_{a}$, that is, at ωt=2π and ωt=5π/2, respectively, after completing a full cycle of the external excitation (i.e., where the magnetic relaxation process has already taken place and the electrodynamics of the HTS coil is within a hysteretic behaviour.)

In summary, starting from the $C_{M}$ model where $J≃\pm J_{c}$, it is possible to observe in a straightforward way, how by analysing the current-density distribution across the thickness of each of the superconducting domains (i.e., within each turn of the 2G-HTS coil), the exact physical nature of the current-density profiles at a local level reveals regions with a concomitant occurrence of positive and negative currents across the thickness of the tape. These both refer explicitly to the self-induced magnetisation currents, which is in good accordance with Bean’s model, and they are enclosed by regions within the magneto-transient history of the applied transport current. For instance, for $I_{a}=0.5I_{c0}$ at ωt=2π, the same amount of positive and negative current-density profiles must exist in order to satisfy the global condition $I_{tr}=0$ and. This implies that starting from the top or bottom edges of the coil towards the centre of each turn, each of the REBCO turns must firstly show a certain amount of current flowing in the positive direction, which then surrounds a weighted region of negative current-density profiles, accounting for the transient history of the applied current, and secondly, a local distribution of the so-called magnetisation currents, whose area decreases as the intensity of the applied current increases, up to its full disappearance at the threshold current condition $I_{tr}=I_{c0}$. In fact, for $I_{tr}<I_{c0}$, the local distribution of current density across the turns of the HTS coil shows a strong dependence on the self-induced magnetisation currents, with a flux-free core visible within the central turns of the superconducting coil. Thus, besides the fact that the intensity of the critical current density for the other material law models in Table 1 cannot be assumed as constant (as is the case for the $C_{M}$ model), all the above-mentioned physical features can be considered as universal, although slight differences between the shape of the flux front profiles have been observed between the different models, which ultimately will lead to the large quantitative differences observed in the definition of the critical current density at each turn of the HTS coil as well as the measured magnetic field and AC losses, which will be discussed in the following subsections.

In more detail, Figure 1 demonstrates how the local dependence of the critical current density with the magnetic field (included through the material laws $K_{M1}$, $K_{M2}$, and $R_{M}$) creates not only a reduction in the critical current density across the width of the coil turns, but also a certain deformation of the flux-front profiles envisaged within the $C_{M}$ model (i.e., the lines of magnetic flux where the slope of *J* across the width of the tape suddenly changes sign). This is especially evident at the transition between the transport current profiles near the tape edges and the enclosed magnetisation currents whose dynamics will first be explained qualitatively. For instance, starting with the current-density profiles at ωt=2π (leftmost pane in Figure 1), it can be observed that although the area with negative profiles of transport current (dark-blue profiles) seems to be smaller than the area with positive profiles (light-yellow profiles), the self-field condition $I_{tr}=0$ is preserved, as the magnitude of $J_{c}$ decreases from the lateral edges of the REBCO layer towards the centre of each of the coil turns.

**Figure 2:**
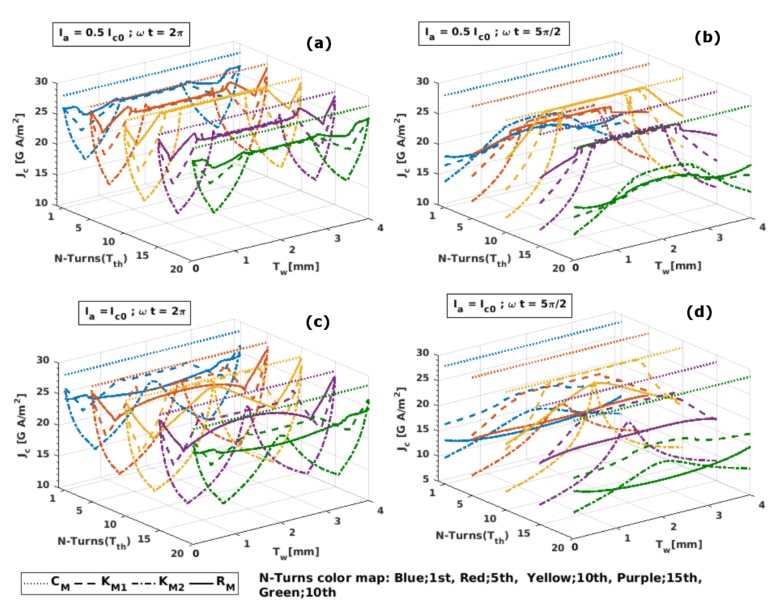
Critical current distribution across the 4-mm-width $\left(T_{w}\right)$ of the 2G-HTS tape measured at the surface of the 1st, 5th, 10th, 15th, and 20th turn $\left(T_{th}\right)$ of the modelled coil (Figure 1), under the different material law conditions in Table 1. In this figure we show (**a**) the results for an applied current of amplitude $I_{a}=0.5I_{c0}$ at the self-field condition ωt=2π and (**b**) the peak transport current condition at ωt=5π/2. Likewise, (**c**) shows the results for an applied current of amplitude equal to the threshold value of the critical current density $I_{c0}$ at ωt=2π and (**d**) at ωt=5π/2.

**Figure 3:**
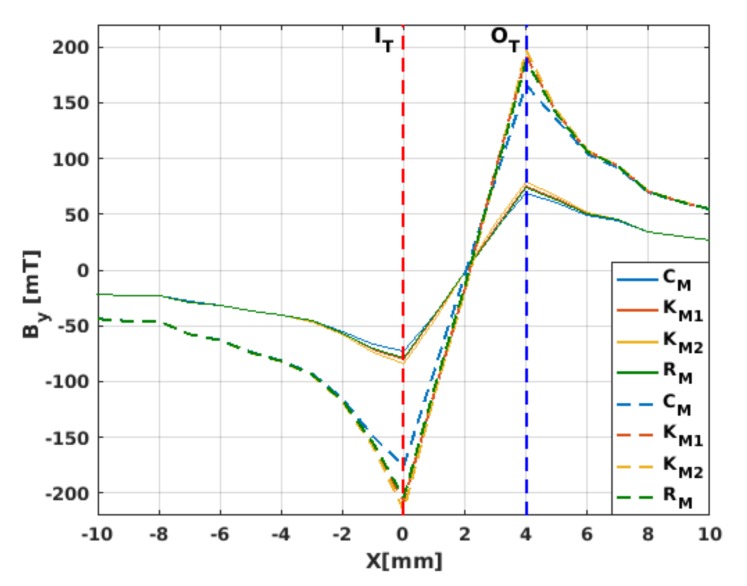
Magnetic field profile over the middle axisymmetric line (*x*-axis) of the ∼ 4-mm coil section (20 turns) displayed in Figure 1 in the direction parallel to the wider surface of the wound 2G-HTS tape, $B_{y}$, measured within and near the coil section for applied currents of amplitude $I_{a}=I_{c0}$ (dashed curves) and $I_{a}=0.5I_{c0}$ (solid curves), at the peak condition ωt=5π/2. The vertical dashed lines at *x* = 0 mm and *x* = ∼ 4 mm respectively refer to the innermost $\left(I_{T}\right)$ and outermost $\left(O_{T}\right)$ turns of the HTS coil.

Notice that, the imbalance in the critical current density across the width of the REBCO layers (see Figure 2) which is caused by the local variance in the magnetic field (see Figure 3) limits then the area of the magnetisation and flux-free cores of the superconducting coil, with the stronger reduction achieved for the empirical $K_{M2}$ model, where the orthonormal components $B_{\|}$ and $B_{⊥}$ are explicitly included. However, such a large reduction in the magnetisation core is not observed in the $R_{M}$ model, whose $J_{c}$ function was directly derived from experimental measurements in 2G-HTS tapes [20], and where θ defines the angle of attack of the magnetic field vector at a local level (see Table 1). In fact, although the $K_{M2}$ and $R_{M}$ models are similar in terms of their mathematical structure, given that the conditions θ=0 and θ=±π/2 in the $R_{M}$ model resemble the parallelism and perpendicularity conditions of the magnetic field assumed in the $K_{M2}$ model, the latter substantially increases the influence of the perpendicular component of the magnetic field when arbitrarily lowering the effect of its parallel component through the empirical microstructure parameter *k*. However, it has already been demonstrated that the actual dependence of $J_{c}$ with the magnetic field vector for the large majority of 2G-HTS tapes can be accurately described by the semi-analytical model derived by Kim [40,41] for fully isotropic REBCO films on the one hand, and on the other hand, for those materials showing a strong magneto-angular anisotropy [20,21], the local components of the magnetic field in $J_{c}$ are averaged by the electron mass anisotropy ratio (γ) of the REBCO layer, which at least for the case of the SCS4050 tape, the distribution of flux front profiles at self field conditions between the $K_{M1}$ and $R_{M}$ models shows nearly the same trend (see Figure 1). This is because strong changes in the critical current density of the SCS4050 tape have been observed only for parallel components of the magnetic field $\left(\theta =0,B=B_{y}\right)$ greater than 50 mT [20], which is a condition seen only for about the first three-to-seven innermost and outermost turns of the HTS coil at moderate-to-high applied transport currents, $I_{tr}\subset \left(0.5I_{c0},I_{c0}\right)$ (see Figure 3). Then, by observing the full penetration condition (i.e., $I_{tr}=I_{c0}$ at ωt=5π/2 in Figure 1), it is possible to see that with exemption of the $C_{M}$ model, the critical current density of the superconducting coil changes across the width and thickness of each of the REBCO turns, which is a phenomenon that was experimentally observed in [13,31]. However, in order to be able to properly see how the $J_{c}$ changes across the different turns of the HTS coil for the different material law models, in the following section we aim to present a more quantitative approach to the above-mentioned observations, which ultimately will be connected to the macroscopic quantities that can be experimentally measured.

### 3.2. Local Profiles of Current Density: A Quantitative Approach

Continuing with our previous discussion, in Figure 2 we present a more detailed picture of how the measurable critical current density over the surface of the individual turns of the HTS coil not only varies along the width of the superconducting tape, but how from the numerical point of view, these predictions can change as a function of the different material laws that can be invoked.

Beginning our discussion with the $C_{M}$ model, it has already been mentioned that the critical current density under this model can only have a single value across the domain of the REBCO layers, as can be seen from the dotted lines in Figure 2. This basically defines the threshold value of the critical current density at self-field conditions, $J_{c0}$, which then has to be lowered by the influence of the magnetic field for magnetically anisotropic superconductors. In this sense, as the intensity of the magnetic field at the innermost and outermost turns of the HTS coil is always greater than the field experienced by the middle turns (see Figure 3), these turns $(T_{th}$ = 1 or 20) will always show the lowest critical current density, regardless of the material law $J_{c}B$. However, as the magnetic field also changes along the width of each of the turns of the HTS coil, with its intensity decreasing from the tape edges towards their geometrical centre; $J_{c}$ increases from the bottom or top corners of the HTS turns (Figure 1), that is, at $T_{w}=0$ and $T_{w}$ = 4 mm in Figure 2, reaching their maximum at the middle distance of the tape width and following the same growing pattern from the innermost and outermost turns towards the centre of the coil, until reaching the magnetic saturation state. This phenomenon can be identified as the plateau in the $J_{c}$ distribution, at the middle turn $\left(T_{th}=10\right)$ for the $K_{M1}$ model (dashed lines) in the peak transport current conditions shown in the right pane plots of Figure 2b,d. Therein, it also can be noticed that with the exception of the innermost and outermost turns of the coil at the peak transport current conditions, the classical $K_{M1}$ model generally overestimates the critical current density across the width of the tape, which is actually one of the main reasons why further approximations for the $J_{c}B$ function have been adopted with time [20,22,33,42,47].

On the other hand, when the computations are performed at the threshold condition for the applied transport current, $I_{tr}=I_{c0}$, emulating the actual conditions for the experimental measurement of $J_{c}$ (see Figure 2d), it can be clearly seen why the empirical and semi-empirical models $K_{M2}$ and $R_{M}$, respectively, can render to exactly the same experimental measurements on long sections of individual 2G-HTS tapes. To notice this, it is necessary to bear in mind that in the common practice for the electrical measurement of the I-V curves, and consequently the magnitude of $J_{c}$, a series of equally distanced voltage taps are all positioned at the middle width of the HTS tape, where the intensity of the $J_{c}$ is maximal. Therefore, by observing the local profile of $J_{c}$ at the central turn of the HTS coil ($T_{th}=10$ at $T_{w}$ = 2 mm), where the *x* component of the magnetic field (perpendicular to the surface of the tape) is null, exactly the same intensity of the critical current density can be obtained from the $K_{M2}$ model (dash-dotted yellow line) and the more general $R_{M}$ model (solid yellow line), although the impact of the parallel component of the magnetic field $\left(B_{y}\right)$ on the local $J_{c}$ profiles for the HTS coil is underestimated as one moves away from the midpoint. This can also be seen by inspecting the last column of subplots shown in Figure 1, where by colour contrast it can be observed how the maximum $J_{c}$ allowed by each model is obtained at the midpoint of the coil section, but also how its magnitude decreases towards the coil periphery, where a larger detriment of its magnitude was seen when assuming the $K_{M2}$ model, which is in good agreement with our previous analysis and current numerical results.

Thus, if the physical quantity aimed to be measured and explained in a superconducting coil is the magnitude of the critical current density for the different turns of the 2G-HTS tape being wound, certain caution must be taken in terms of the adequate selection of a material law that describes the complete magneto-angular anisotropic properties of the REBCO layer (from the theoretical perspective), and the correct alignment of the voltage taps in the experimental measurement. Otherwise, differences of up to about 50% between the theoretical and experimental measurements can arise at the innermost and outermost turns of the coil. However, if the critical current density is rather measured by magneto-optical imaging techniques [53,54,55,56], where it is possible to observe the full dynamics of the critical current density along the width of the tape surface $\left(T_{w}\right)$, for the self-field and partial penetration conditions of the HTS coil (i.e., at the transport current condition $I_{a}=0.5I_{c0}$ and ωt=2π in Figure 2a), an outstanding resemblance with the experimental measurements reported in [56] was found when the magnetic-field dependence of $J_{c}$ was considered. In fact, in this paper we confirm that during the initial flux penetration, the actual current distribution inside the YBCO tape does not show any plateau-like feature, as would be expected from the $C_{M}$ model, but instead it develops strong peaks near the tape edges, as determined by the Kim’s hypothesis $\left(J_{c}\propto B\right)$ contained within the $K_{M1}$, $K_{M2}$, and $R_{M}$ models. However, further investigation of the magnetisation properties of single tapes under applied magnetic fields at different orientations (out of the scope of this paper) will need to be conducted to determine if whether the complete validity of one or another model needs to be established beyond the broad success that the $R_{M}$ model has already achieved over a large set of commercially available 2G-HTS tapes [20,21].

### 3.3. Magnetic Field Ratio within Different Material Laws

**Figure 4:**
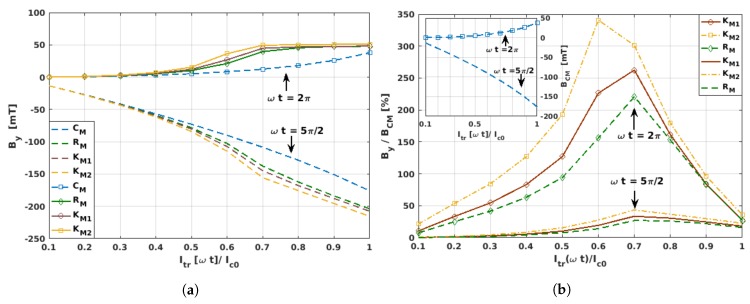
(**a**) Magnetic field component $B_{y}$ at the middle point of the external surface of the innermost turn of the 2G-HTS coil as a function of the applied transport current $I_{tr}\left[\omega t\right]$ at the self-field (ωt=2π) and peak transport current (ωt=5π/2) conditions, and the different material laws presented in Table 1. (**b**) The relative percent ratio between the different material laws, taking as a reference the $B_{y}$-field obtained within the critical-state-like-model $C_{M}$ (inset).

**Figure 5:**
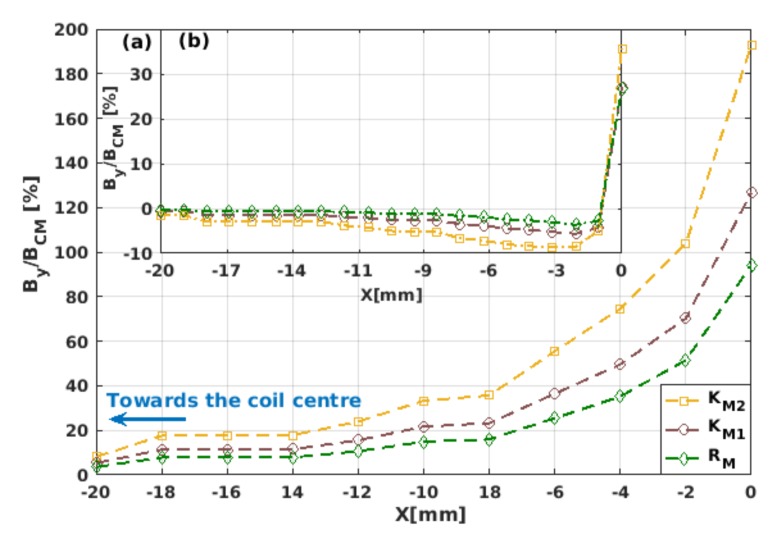
Magnetic field component $B_{y}$ along the middle axisymmetric line (*x*-axis) of the 2G-HTS coil calculated within the Kim-based approaches $K_{M1}$, $K_{M2}$, and $R_{M}$ (Table 1) as a percentage function relative to the field predicted by the isotropic $C_{M}$ model. The measured field is presented from the innermost turn of the coil at 0 mm (see Figure 3) towards the coil centre at the self-field condition ωt=2π, when the applied current has an amplitude of (**a**) $I_{a}=0.5I_{c0}$ (i.e., with the central turns of the HTS coil partly magnetised (see Figure 1)), and (**b**) $I_{a}=I_{c0}$ (inset), that is, with the HTS coil fully saturated by the transport current and no magnetisation currents.

After having presented a critical discernment of the main physical features that could be observed by the measurement of the local current density in a superconducting coil under the framework of different material laws (Table 1), in this section we present a systematic analysis of how the intensity of the magnetic field component $B_{y}$ over and near the surface of the innermost turn of the HTS coil (see Figure 4 and Figure 5, respectively) changes as a function of the applied transport current in the self-field condition (ωt=2π), and in the peak transport current condition $I_{tr}=I_{a}$ at ωt=5π/2.

Note that the apparently arbitrary position on which we decided to display the magnetic field profiles (Figure 4), that is, over the external surface of the innermost coil turn, is indeed the best case for evincing the relative differences between the material laws in Table 1, as negligible changes on the magnetic field were found at the centre of the coil former between all these models (see Figure 5). Thus, if in an actual experimental setup the magnetic field is to be measured at the centre axis of the HTS coil, our numerical results allow us to conclude that the main factor in deciding what material law to use in the computational modellers will not be the dependence of the critical current density with the vector of magnetic field (as in the $K_{M1}$, $K_{M2}$, and $R_{M}$ models), but the extent of the computational time demanded by each of the models. In this sense, it is worth mentioning that, on average, the $C_{M}$ model always gave the fastest numerical solution, which therefore makes this material law the most suitable model, as long as the interest of the modeller is in targeting the aforementioned experimental conditions.

Evidently, the difference between the magnitude of the magnetic field predicted by the critical-state-like model $\left(C_{M}\right)$ and the Kim-based models ($K_{M1}$, $K_{M2}$, and $R_{M}$) is larger as the intensity of the applied current increases (see Figure 4). However, through the macroscopic measurement of $B_{y}$, a remarkable difference between the $C_{M}$ and the Kim-based models can be observed as a function of the applied transport current for the self-field (ωt=2π) and peak transport current (ωt=5π/2) conditions, which we found to be intrinsically connected with the dynamics of the flux front profiles described in Section 3.1. In this sense, in Figure 4a it can be seen that for low intensities of the applied current, $I_{tr}≲0.4I_{c0}$, nearly no difference was obtained in the magnitude of the derived field, meaning that no appreciable difference would be seen between the dimensions of the local flux front profiles between the different material law models. For this reason, the smallest amplitude of the transport current where the local profiles of current density have been shown in this paper is just $I_{a}=0.5I_{c0}$ (Figure 1). However, for moderate intensities of the applied current, ∼$0.4I_{c0}≲I_{a}≲0.8I_{c0}$, the magnetic field curves obtained from the Kim-based models rapidly deviate from the isotropic $C_{M}$ model, all showing a “kink-like” curvature which separates the physical behaviour between what we called moderate and high applied currents. More specifically, the range of high applied currents is characterised by the magnetic saturation of the superconducting coil, and therefore there is a plateau in the measurement of $B_{y}$ at ωt=2π, meaning that for applied currents $I_{tr}\geq 0.8I_{c0}$, none of the turns of the superconducting coil can exhibit a flux-free core with local condition J=0. On the other hand, the physics of the HTS coil at moderate currents are characterised by transient states, where the Lorentz force between the local profiles of transport current and the induced magnetisation ones is sufficiently strong to reduce the dimensions of the flux-free core by the flux-creep of magnetisation currents, which is added to their own consumption by the extrinsic condition of applied transport current. For a more detailed explanation of this cumbersome phenomenon, which in simple terms corresponds to the actual response of any type-II superconductor to the concomitant action of a magnetic field and a transport current, we encourage the reader to follow references [57,58,59,60], where the physical richness of the full dynamics of flux-front profiles in simplified geometries has been exploited.

By adopting a more quantitative approach to illustrate the differences between the use of one or another material law, in Figure 4b we show the percent ratio between the Kim-based material laws and the $C_{M}$ model (Table 1), where the larger differences were found at the self-field condition ωt=2π. Therein, a clearer picture of the change of the flux creep dynamics between fully isotropic samples which obey the $C_{M}$ model and those with the magneto-anisotropic properties introduced by the Kim-based models can be seen. For instance, note that in the low-intensity regime $\left(I_{tr}≲0.4I_{c0}\right)$, the increment of the $B_{y}/B_{CM}$ curves primarily obeys a linear function. This means that the physical mechanism leading to the flux creep in the coil turns during the low-current regime is mainly the linear consumption of the magnetisation currents caused by the global condition of transport current, but that at the same time and from local dynamics, it shows that the amount of resulting magnetisation currents inside of each of the coil turns remains nearly the same for all of them. On the other hand, two different phenomena can be recognised for the so-called moderate-intensity regime $\left(0.4I_{c0}≲I_{tr}≲0.8I_{c0}\right)$ [27]. Firstly, the field $B_{y}/B_{CM}$ rapidly increases up to a maximum value, at which the relatively spatial balance between the magnetisation currents and the transport currents (per turn of the coil) cannot be maintained. This leads to a transformation of the flux front profile from a square-like shape towards an elliptical shape (see Figure 1), although during this regime the dimensions of the flux-free core at the centre of the coil are still unaltered. Secondly, when the $B_{y}/B_{CM}$ curve follows a rapid drop as shown in Figure 4b, the flux creep not only occurs at the positions where there is a consumption of the induced magnetisation currents by the applied transport currents (i.e, where the outer flux front profile can be evinced), but also at the positions where the innermost magnetisation currents are pushed by the superconducting Lorentz force towards the centre of the coil. This reduces the dimensions of the flux-free core, which was previously unaltered. This phenomenon continues until a current level where no evidence of a flux-free core can be observed $\left(I_{tr}\approx 0.8I_{c0}\right)$, after which a linear behaviour of $B_{y}/B_{CM}$ is achieved. Thus, having previous knowledge of the electromagnetic behaviour of an HTS coil within the limits of the $C_{M}$ model can be considered as a practical approach, not only because this model is more computationally affordable, but because when comparing the actual measurement of the $B_{y}$ profile near the innermost or outermost turns of the coil with the predicted $B_{CM}$ values, a simple idea of how the local profiles of current density are can be extracted without using more sophisticated experimental techniques. Likewise, in good agreement with our former analysis, for the flux front profiles and the local distribution of current density as a function of the material law, all Kim-based models predict an increment on the intensity of the magnetic field over the surface of the innermost tape of about 2 to 3 times the magnetic field predicted by the $C_{M}$ model, with the $K_{M}$ model somehow acting as a mean-field approach for the magneto-angular anisotropic characteristics of REBCO tapes. In this sense, although we demonstrated that the $C_{M}$ model can be considered as a valid approach when the magnetic field is measured at the centre of the coil former, and also that it can provide some means to elaborate the dynamics of the flux front profiles for magnetically anisotropic materials, we now know that the $C_{M}$ model cannot be quantitatively accounted if the magnetic field is to be measured near the coil turns. Therefore, instead of the $C_{M}$ model, the simplified Kim’s model $K_{M1}$ can be assumed if a relative tolerance between the experimental and numerical results of ∼25% is accepted. Otherwise, and under the expense of possibly increasing the computing time by a factor of 1.5–2, it would be necessary to appeal to a more general approach such as the $R_{M}$ model.

### 3.4. AC Losses

**Figure 6:**
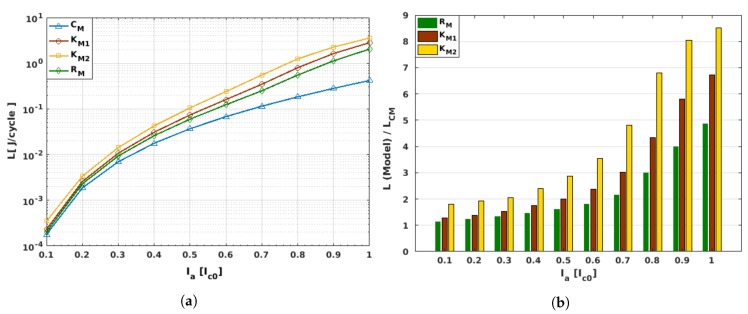
Presented as a function of the amplitude of the applied transport current, $I_{a}$, and the material law models introduced in Table 1, in this figure we show (**a**) the predicted AC losses in Joules/cycle for a 20-turn SCS4050 racetrack coil, and (**b**) the relative difference between the Kim-based anisotropic models ($R_{M}$, $K_{M1}$, and $K_{M2}$) and the magnetically isotropic $C_{M}$ model.

Finally, in this section we present our calculated AC loss curves for the benchmarked 20-turn SCS4050 racetrack coil, where the differences between the hysteresis losses that could be predicted by the use of a magnetically isotropic model (e.g., the critical-state-like model, $C_{M}$) and the respective Kim-based magneto-anisotropic models $R_{M}$, $K_{M1}$, and $K_{M2}$ (see Table 1) are highlighted (Figure 6). Here, it is worth mentioning that although the $C_{M}$ model predicts all the macroscopic electromagnetic characteristics of the type-II superconductors well, its validity relies mainly on the qualitative figures of the model, rather than on the quantitative scope added by the Kim-based models. In this sense, in Figure 6a, note how the $C_{M}$ model generally underestimates the actual AC-losses of the superconducting system, similarly to our previous discussions about the intensity of the magnetic field created by the superconducting coil at different excitation conditions. In fact, the case of a racetrack coil is in good agreement with our previous findings about flux front dynamics and the local distribution of profiles of current density, in which for the low current range $\left(I_{a}\leq 0.4I_{c0}\right)$, all the material law models render to nearly the same hysteretic loss, with relative differences no greater than about twice the losses predicted by the $C_{M}$ model. This factor might actually seem substantial for some readers, but the AC losses of a type-II superconducting coil can increase by orders of magnitude by just increasing the intensity of the applied current, and in this sense an increment of about two times the estimated losses by the $C_{M}$ model can be considered, within the ratio of tolerance between the experimental and numerical measurements. However, for moderate-to-high intensities of the applied current, $\left(I_{a}≳0.4I_{c0}\right)$, we already established that the impact of the magneto-angular anisotropy of the REBCO layer is very significant. Therefore, the difference with the predicted losses by the $C_{M}$ model augment significantly as $I_{a}$ approaches its critical value, $I_{c0}$ (see Figure 6b). For instance, taking as an example this threshold condition for the applied transport current, $I_{a}/I_{c0}=1$, the hysteresis losses of the racetrack coil predicted by the $C_{M}$ model are $L_{CM}=0.4193$ J/cycle, whilst for the fully magneto-anisotropic model, $R_{M}$, the predicted losses are about 4.86 times greater than this value (i.e., $L_{RM}≃2.039$ J/cycle). Likewise, if the empirical $J_{c}$-function for the $K_{M2}$ material law is applied, a maximum increment of about 8.5 times the $L_{CM}$ losses are predicted, $L_{KM2}≃3.57$ J/cycle, as a consequence of the increment in the magnitude of the critical current density, caused by the arbitrary reduction of the parallel component of the magnetic field in the function $J_{c}\left(B_{\|},B_{⊥}\right)$, which was assumed by the $K_{M2}$ model (see Table 1). However, once again the most classical and generic approach of the $J_{c}\left(B\right)$ function introduced by Kim, where only spatial but not angular anisotropy is considered (i.e., the $K_{M1}$ model), serves as an average model between the most tailored approaches $K_{M2}$ and $R_{M}$, with $L_{KM2}≃2.816$ J/cycle at $I_{a}=I_{c0}$. Thus, although very strong differences in the predicted energy losses of superconducting racetrack coils could be envisaged by the use of different material laws, especially if the magnetic anisotropic properties of the REBCO layer are considered, we can conclude from this study that if the actual material law governing the $J_{c}$ properties of the 2G-HTS tape is not known (e.g., the $R_{M}$ model, where most of the microstructural parameters have already been connected with other physical quantities), then it is advisable to just consider the classical Kim’s model for numerical purposes, as long as a tolerance window of about ±28% difference between the experimental and numerical hysteresis losses is allowed.

## 4. Conclusions

Although many experimental measurements can be at least qualitatively explained by different E-J power law models, for a few decades, some questions about the general validity of the E-J power law remained unsolved. For instance, as the two main criteria for deciding what material law to use in the numerical modelling of a superconducting device are the reproduction of specific experimental evidence (either qualitatively or quantitatively) and the affordability of the computation (due to the usually large demand on memory and processing power that finite element method (FEM)simulations require, especially with the large aspect ratio of geometries such as those implied by the 2G-HTS tapes), it is natural to wonder if the E-J power law invoked by a researcher is a simple artificial function that has been used to match a single piece of experimental evidence. Is the assumed material law model sufficiently valid and well-supported to reproduce all other macroscopical electromagnetic quantities within a more quantitative perspective? Or, in fact, could we in a practical manner use a simpler material law that is capable of accounting for the different electromagnetic phenomena of type-II superconductors, regardless of whether the superconducting material is known to exhibit magneto-anisotropic properties, as is the case for the majority of 2G-HTS tapes? Thus, in an attempt to answer these questions, in this paper we analysed how the selection of different material laws in the numerical modelling of superconducting coils can strongly influence the accurate estimation of macroscopically measurable physical quantities such as the critical current density per coil turn, the magnetic field near the coil armature, and the accounting of the hysteresis losses per cycle, which were all considered under self-field current conditions.

Four different material laws were considered in this study, where a clear impact of the magneto-anisotropic properties of 2G-HTS tapes was disclosed through the direct comparison between an isotropic critical-state-like model, $C_{M}$, and different versions of the so-called Kim-based models: $K_{M1}$, $K_{M2}$, and $R_{M}$ in Table 1. These are amongst the most prevalent E-J power law models for 2G-HTS tapes, all validated up to certain extent under different experimental conditions [20,23,33,39,40,47]. We found that although each of these material laws allows a proper qualitative description of the electromagnetism of superconducting coils, substantial quantitative differences were found between their predictions under common experimental conditions, which ultimately, from a purely computational perspective, can help modellers to make a decision on what material law could be more suitable when time and computing power are both limited. In this sense, we concluded that when the physical quantity to be measured is the critical current density turn-by-turn by I-V measurements, certain caution must be taken when compared with the numerical results (Figure 2), as depending on the positioning of the voltage taps, the local magneto-angular anisotropy of the superconducting tape can lead to deviations of up to 50% between the theoretical and experimental measurements. Moreover, if the $C_{M}$ model is assumed, then no difference in $J_{c}$ for the different turns of the superconducting coil could be predicted. On the contrary, if a Kim-based model is invoked, then the local variation of the critical current density across the surface of the superconducting tape could be visualised in good agreement with the magneto-optical imaging observations reported in [56]. However, if the intensity of the magnetic field at the central axis of the 2G-HTS coil is the physical quantity of focus (Figure 3), then the simplicity and minimum computing time that can be achieved with the $C_{M}$ model makes this material law the best option, as no difference was obtained when it was compared with the prognostics of the Kim-based models. Nonetheless, the situation is different if the intensity of the magnetic field near the innermost or outermost turns of the superconducting coil is the desired measurand (Figure 4), as at these locations, local changes in the distribution of current-density profiles can be macroscopically evinced by analysing the $B_{y}$ behaviour at low, moderate, and high transport currents. In this sense, under this scenario, the simplified Kim’s model $K_{M1}$ can be chosen as the most suitable candidate for the numerical modelling of the superconducting properties, as long as a relative tolerance between the experimental and numerical results of ∼25% is accepted, and if the expected increase in the computing time cannot be afforded when a more “tailored” approach such as the $R_{M}$ model is invoked.

Remarkably, when comparing the $B_{y}$ curve of the Kim-based models with the one derived by the $C_{M}$ model, over the surface of the innermost turn of the superconducting coil, we obtained an extricable mean for the experimental determination of the magnetic saturation state of the entire coil at self-field conditions and zero transport current (ωt=2π), which is shown in the form of a $B_{y}/B_{CM}$ plateau for high amplitudes of the applied transport current. Besides, when the study of the superconducting coil focuses on the measurement or estimation of the hysteresis losses, we found that despite the expected underestimation of the AC losses by the $C_{M}$ model, for low intensities of the applied current $\left(I_{a}\leq 0.4I_{c0}\right)$, all the magneto-anisotropic models led to nearly the same results, with a relative difference of maximum twice the losses expected by the $C_{M}$ model, which is not necessarily seen to be as large as the hysteretic losses themselves, and can change in orders of magnitude as the intensity of the applied current increases. Nevertheless, for moderate-to-high intensities of the applied current $\left(I_{a}≳0.4I_{c0}\right)$, the impact of the magneto-angular anisotropy of the superconducting tape was very significant, with differences found between ∼3 to ∼8 times the estimated losses of the isotropic $C_{M}$ model. Evidently, if the computing time and power are not a matter of concern for the modeller of superconducting machines, the best option for choosing the material law for quantitative purposes would then be to select one where the majority of the microstructural parameters for the superconducting tape can or have already been determined by experimental measurements, as is the case of the $R_{M}$ model. However, if the used 2G-HTS tape is one where the $J_{c}\left(B,\theta \right)$ is unknown, then we can conclude that the use of the classical Kim’s model would be the most advisable choice, if the numerical modellers and experimentalists bear in mind a relative tolerance of ∼28% in the estimation of the AC losses.

## Figures and Tables

**Table 1:** Material law models and related variables considered within the E-J power law formulation for the rare-earth barium-copper oxide (REBCO) material in Equation (1).

Model	Legend	Simplified Description	Material Law $J_{c}=$	Microstructure Parameters
Critical-State (CS)-Like model [23,39]	$C_{M}$	$J=\pm J_{c}$	$J_{c0}$	$J_{c0}=2.85\times 10^{10}$ A/m ${}^{2}$ [42]
Kim’s Model [40]	$K_{M1}$	$J_{c}\left(B\right)$	$\frac{J_{c0}}{{\left(1+\frac{|B|}{B_{0}}\right)}^{\alpha }}$	$B_{0}=240$ mT α=1.5 [22,42]
Kim-Like model [47]	$K_{M2}$	$J_{c}\left(B_{\|},B_{⊥}\right)$	$\frac{J_{c0}}{{\left(1+\frac{\sqrt{k^{2}|B_{\|}{|}^{2}+{\left|B_{⊥}\right|}^{2}}}{B_{0}}\right)}^{\alpha }}$	$B_{0}=42.65$ mT α=0.7 k=0.29515 [33,42,47]
Magneto-Angular Anisotropy Model [20]	$R_{M}$	$J_{c}\left(B,\theta \right)$	$\frac{J_{c0}}{{\left(1\;+\;\epsilon_{\theta }{\left(\frac{|B|}{B_{0}}\right)}^{\beta }\right)}^{\alpha }}$	$\epsilon_{\theta }=\sqrt{\gamma^{-1}\sin^{2}\left(\theta \right)+\cos^{2}\left(\theta \right)}$ $B_{0}=240$ mT, α=1.5 β=1, γ=5.02 [20,22,42]

## Abbreviations

The following abbreviations are used in this manuscript:

2D	Two-dimensional
2G-HTS	Second-generation of high-temperature superconductor/superconducting
AC	Alternating current
CS	Critical state
DC	Direct current
FEM	Finite element method
PDE	Partial differential equation
REBCO	Rare-earth barium-copper oxide
YBCO	Yttrium barium copper oxide
